# Supplementary material for: Support-tuned iridium reconstruction with crystalline phase dominating acidic oxygen evolution
Source: Nat Commun. 2025 Sep 1;16:8164. doi: 10.1038/s41467-025-63541-9 (PMC12402471; doi:10.1038/s41467-025-63541-9)
Supplement: Supplementary file 1 — Supporting Information [file 41467_2025_63541_MOESM1_ESM.pdf]

Supporting Information (SI) for

Support-Tuned Iridium Reconstruction with Crystalline Phase Dominating Acidic Oxygen Evolution

Kexin Zhang,[1, †] Xiao Liang,[1, †] Yucheng Wang,[2] Yongcun Zou,[1] Xiao Zhao,[3] Hui Chen[1, *] and Xiaoxin Zou[1, *]

[1] State Key Laboratory of Inorganic Synthesis and Preparative Chemistry, College of Chemistry, Jilin University, Changchun 130012, China

[2] State Key Laboratory of Physical Chemistry of Solids, College of Chemistry and Chemical Engineering, Xiamen University, Xiamen 361005, China

[3] Key Laboratory of Automobile Materials of MOE, School of Materials Science and Engineering, Jilin University, Changchun 130012, China

[†] K. Zhang and X. Liang contributed equally to this work

* E-mail: chenhui@jlu.edu.cn; xxzou@jlu.edu.cn

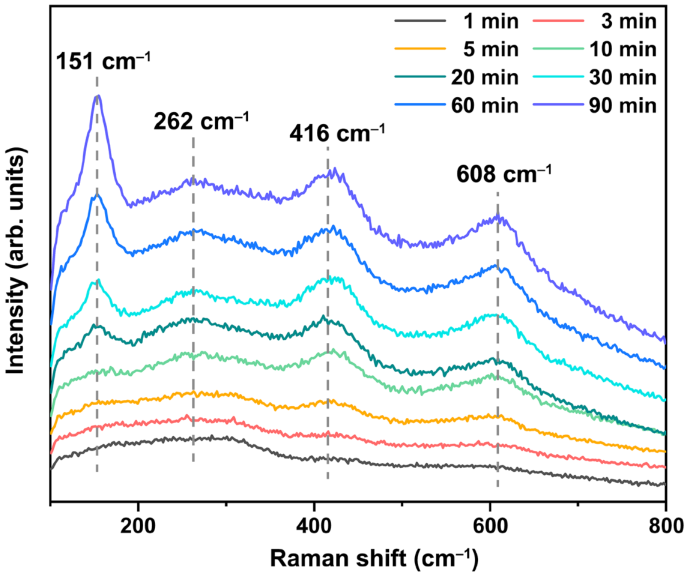

Supplementary Figure 1. Quasi-in-situ Raman spectroscopy of Ti nanospheres heated directly in ethylene glycol under air conditions. The signal of Ti oxide gradually increases over time, confirming the progressive oxidation of Ti within the reaction system.

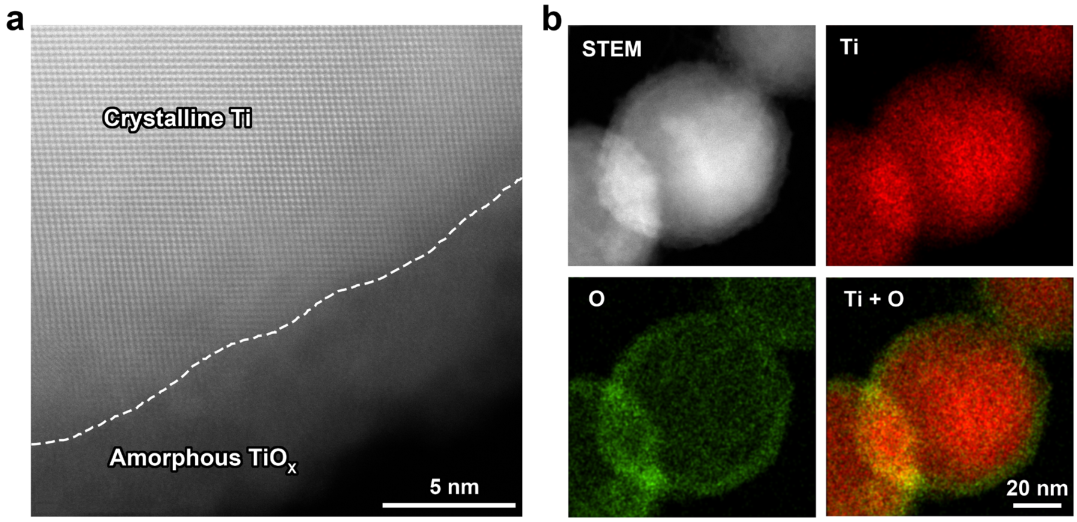

Supplementary Figure 2. (a) The aberration-corrected HAADF-STEM images of TiO_x@Ti. (b) Elemental mapping images of TiO_x@Ti. TiO_x@Ti exhibits a distinct shell@core structure, with the core consisting of crystalline Ti and the shell comprising an amorphous TiO_x layer approximately 5 nm thick. Elemental mapping confirms the homogeneous distribution of oxygen within the shell.

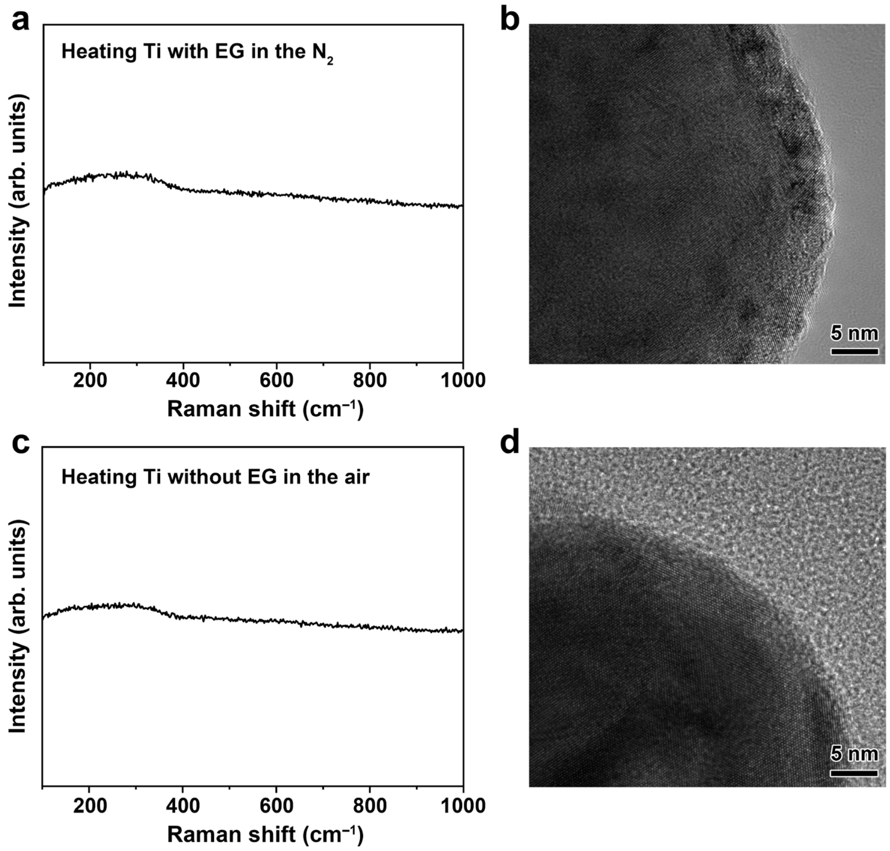

Supplementary Figure 3. (a) Raman spectroscopy and (b) TEM image of Ti nanospheres heated at 180°C with EG in the N_2 indicate that under these conditions, no oxide forms on the Ti surface (c) Raman spectroscopy and (b) TEM image of Ti nanospheres heated at 180°C without EG in the air indicate that under these conditions, no oxide forms on the Ti surface. These results demonstrates that oxygen is the direct source for the formation of TiO_x layer on Ti nanospheres, and EG promotes the reaction between oxygen and Ti nanospheres.

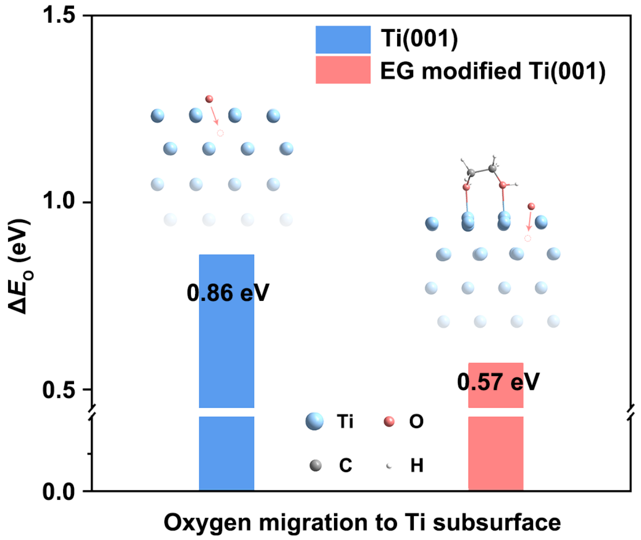

Supplementary Figure 4. Adsorption energy change of oxygen (ΔE_O) from surface to subsurface on clean Ti(001) and EG modified Ti(001). The inset shows the structural models of oxygen atom adsorption on the Ti(001) and EG modified Ti(001) surfaces, with arrows indicating the migration process of the oxygen atom towards the subsurface. It shows that a smaller ΔE_O for EG modified Ti(001) indicates that oxygen migration into the subsurface is thermodynamically more favorable, reflecting the adsorption of EG on Ti surface enhances the diffusion of oxygen into Ti lattice.

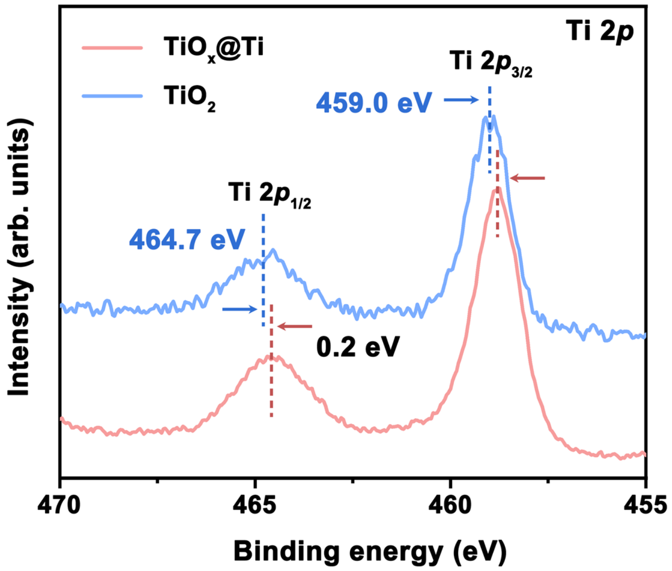

Supplementary Figure 5. Ti 2*p* XPS spectrum of TiO_x@Ti and TiO_2.

Precisely determining the stoichiometric parameter x within the amorphous TiO_x layer remains challenging. Nevertheless, surface-sensitive XPS analysis of the Ti 2*p* region offers valuable insight. Compared to commercial TiO_2, the Ti 2*p* peaks for TiO_x@Ti exhibit a distinct 0.2 eV shift towards lower binding energies. This shift clearly indicates that x is less than 2.

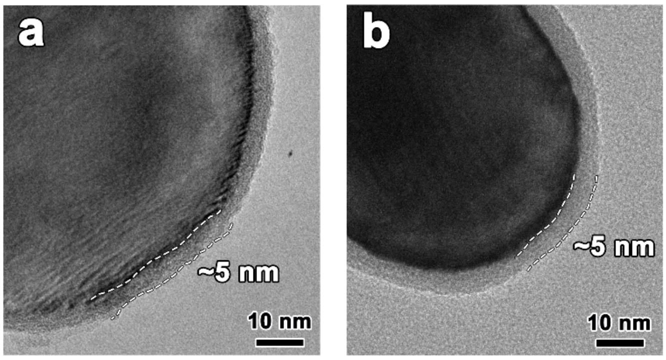

Supplementary Figure 6. TEM images of nano-Ti powder after reaction in ethylene glycol at 180°C in air for (a) 2 hours and (b) 5 hours.

The thickness of the amorphous TiO_x shell is not controllable under the synthesis conditions explored. This is evidenced by comparative experiments where pure nano-Ti powder was reacted in ethylene glycol alone for 2 hours and 5 hours (**Supplementary Figure 6**). Despite the differing reaction times, the resulting TiO_x shell thickness consistently reached ~5 nm. This consistency indicates that the oxidation process reaches a thermodynamic limit, forming a passivating TiO_x layer that prevents further bulk oxidation of the underlying Ti metal.

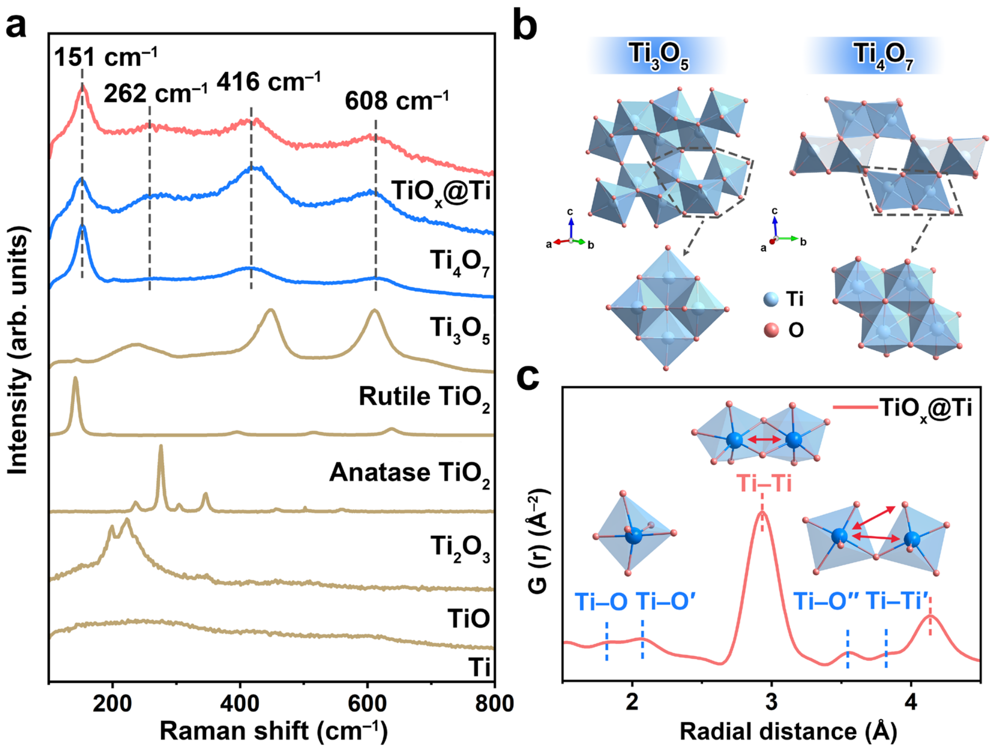

Supplementary Figure 7. (a) Raman spectroscopy of TiO_x@Ti and several Ti oxides. (b) Crystal structure of Ti_3O_5 and Ti_4O_7. (c) X-ray atomic pair distribution function of TiO_x@Ti. The inset displays structural models of a single TiO_6 octahedron, two edge-sharing TiO_6 octahedra, and two corner-sharing TiO_6 octahedra.

Raman spectroscopy of TiO_x@Ti and several Ti oxides were collected. As shown in **Supplementary Figure 7a**, the TiO_x@Ti exhibits four characteristic peaks of the Ti−O bonds at 151 cm^{-1}, 262 cm^{-1}, 416 cm^{-1}, and 608 cm^{-1}, which are assigned to Ti_3O_5 or Ti_4O_7[1,2]. The result indicates that TiO_x layer contains small Ti_3O_5 or Ti_4O_7-like clusters that are different to the thermodynamically stable rutile and anatase TiO_2. The crystal structures of Ti_3O_5 and Ti_4O_7 are shown in **Supplementary Figure 7b**, both of which exhibit notable edge-sharing Ti−O octahedra. The local structure of TiO_x layer is further supported by the X-ray atomic pair distribution function (**Supplementary Figure 7c**). Strong peaks around 2.95 Å and 4.20 Å are ascribed to the Ti−Ti distance in metallic Ti, while other weaker peaks correspond to contributions from Ti-based oxides. The characteristic peaks at 3.56 Å and 3.82 Å cannot be attributed to rutile or anatase TiO_2, but instead align well with Ti_3O_5 or Ti_4O_7. Overall, these results demonstrates that the local structure of TiO_x layer on Ti surface is composed of Ti_3O_5 or/and Ti_4O_7-like clusters.

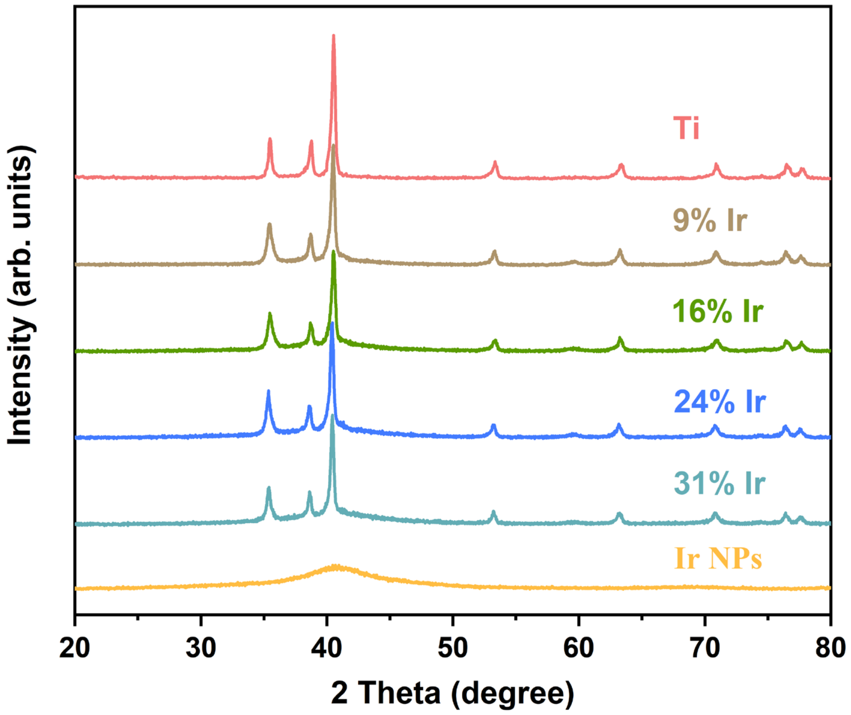

Supplementary Figure 8. XRD patterns of Ti nanospheres, Ir NPs and Ir/TiO_x@Ti with Ir content of 9 wt%, 16 wt%, 24 wt%, and 31 wt%.

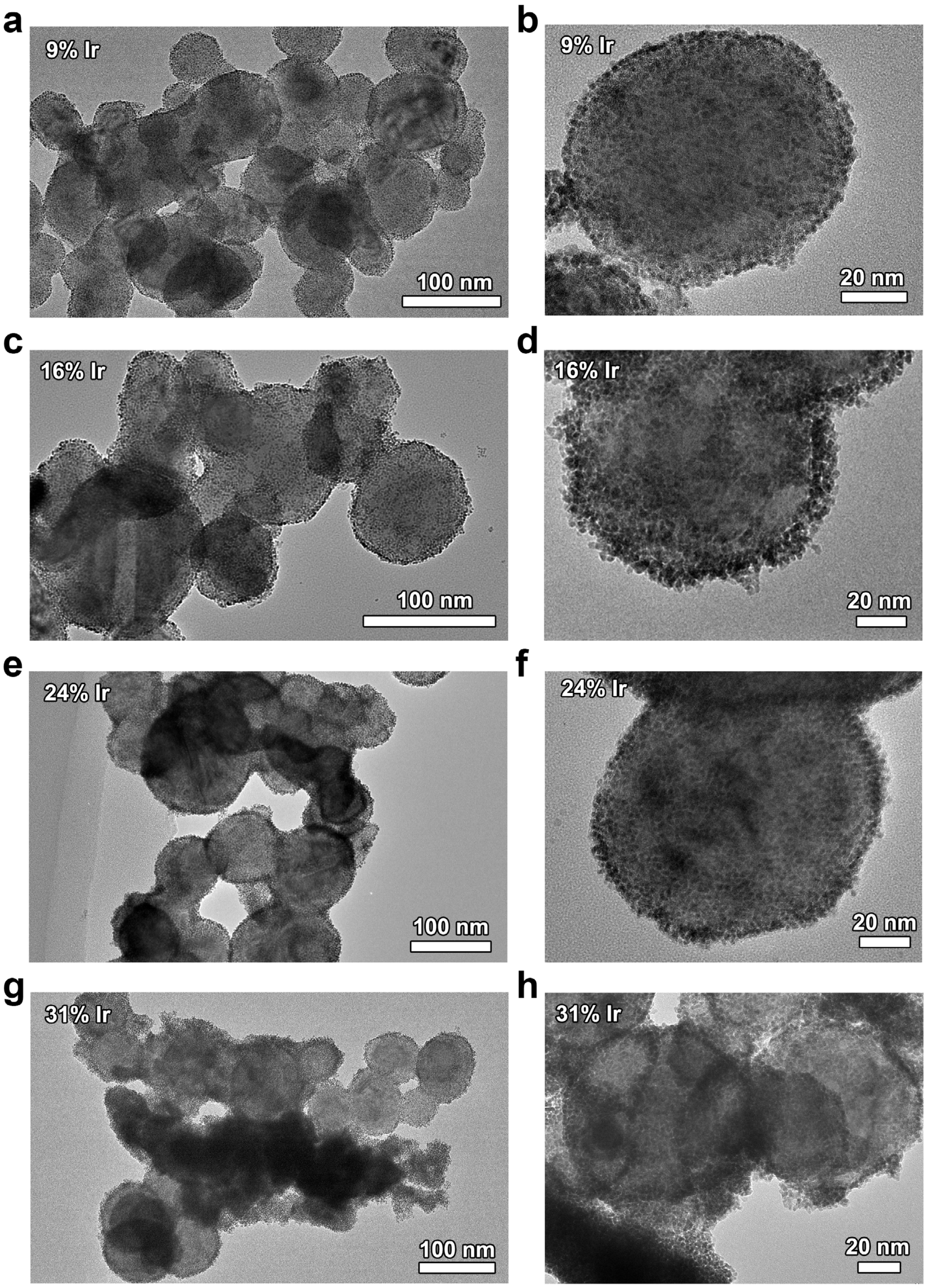

Supplementary Figure 9. TEM images of Ir/TiO_x@Ti with Ir content of (a, b) 9 wt%, (c, d) 16 wt%, (e, f) 24 wt%, and (g, h) 31 wt%.

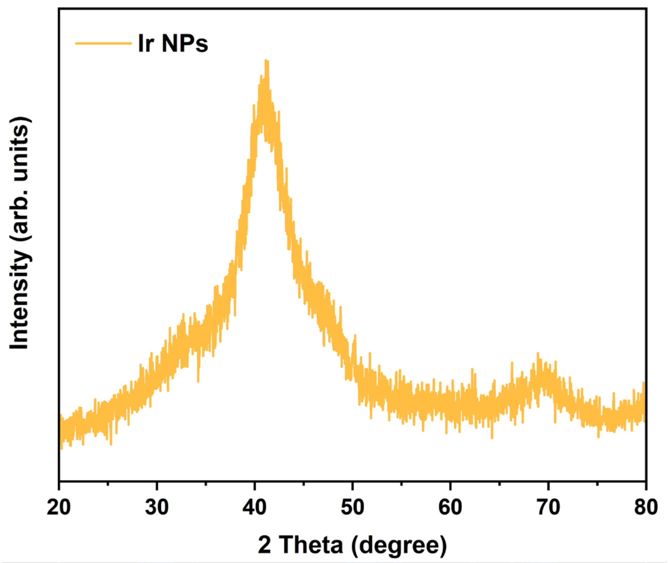

Supplementary Figure 10. XRD pattern of Ir NPs.

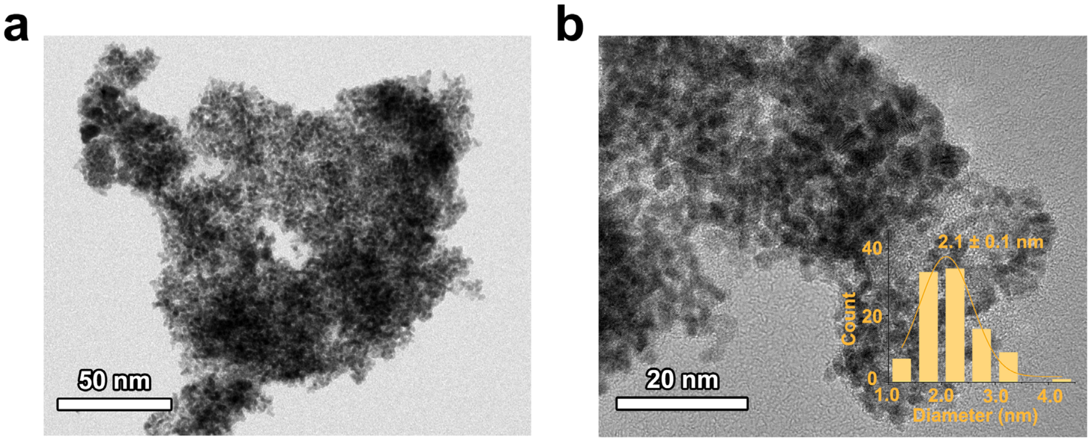

Supplementary Figure 11. (a, b) TEM images of Ir NPs. The inset displays the size distribution histogram obtained from 100 particles

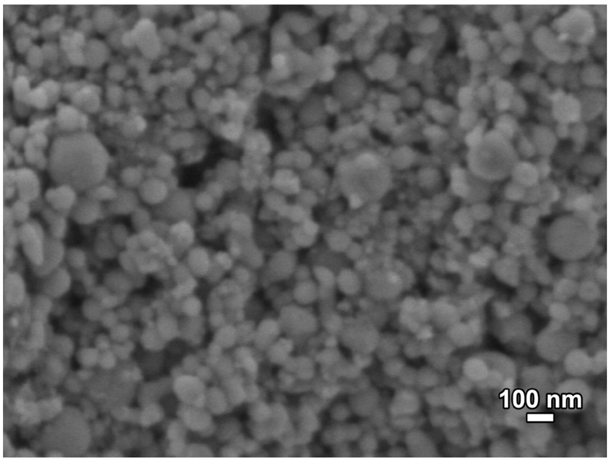

Supplementary Figure 12. SEM image of Ir/TiO_x@Ti.

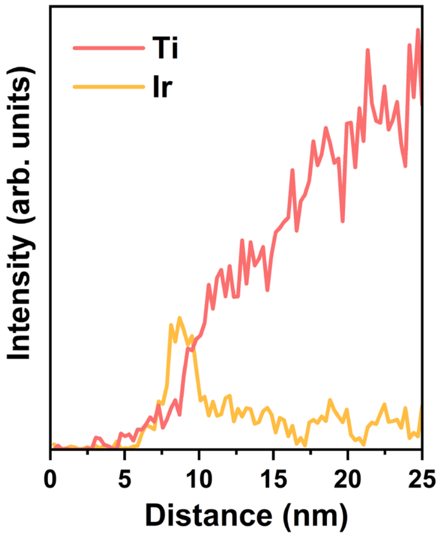

Supplementary Figure 13. EDX line scan profile of Ti and Ir elements in Ir/TiO_x@Ti.

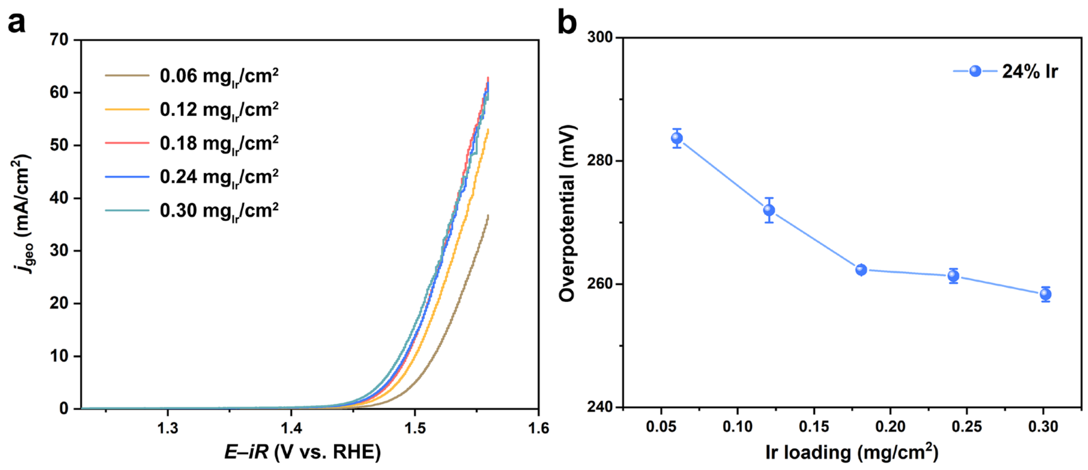

Supplementary Figure 14. (a) The polarization curves for OER of 24% Ir/TiO_x@Ti with varying Ir loading of on glassy carbon electrode at a scan rate of 1 mV/s. j_{geo} represents the current density normalized to the geometric electrode area, and $E–iR$ denotes the potential after 85% iR compensation. (b) The correlation of Ir loadings on glassy carbon electrode with overpotentials at 10 mA/cm^2 current density for 24% Ir/TiO_x@Ti sample. Error bars are drawn based on the standard deviations of three measurements.

The OER overpotentials for 24% Ir/TiO_x@Ti exhibit linear decrease with increasing Ir loading up to 0.18 mg/cm^2, suggesting a linear interrelationship between the number of accessible Ir sites and Ir loading. When the loading further increased, OER overpotential remains relatively constant, because the working electrode is fully coated with the 24% Ir/TiO_x@Ti catalyst. Hence, the Ir loading on the glassy carbon electrode is 0.18 mg/cm^2 in subsequent electrochemical tests.

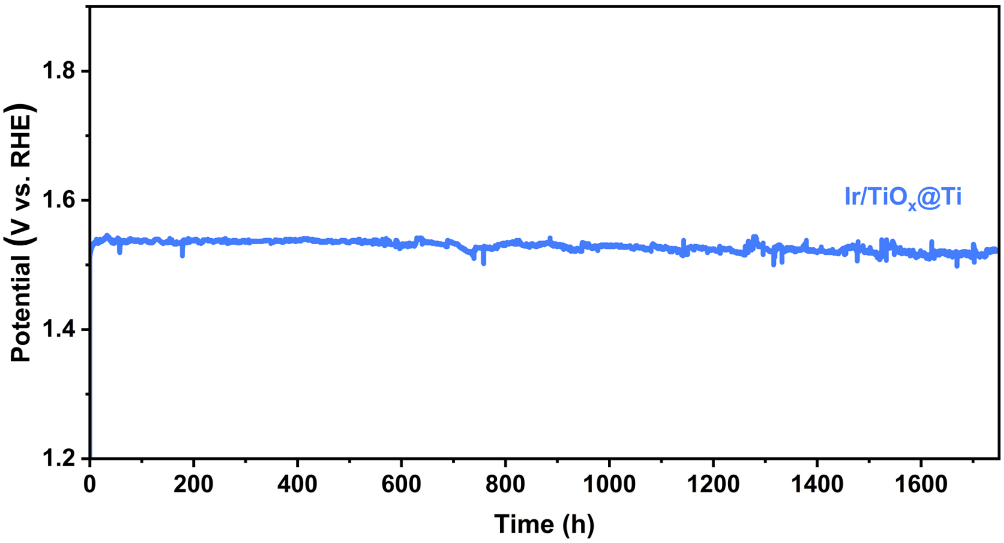

Supplementary Figure 15. The chronopotentiometry curve of Ir/TiO_x@Ti at 10 mA/cm^2 current density without iR compensation.

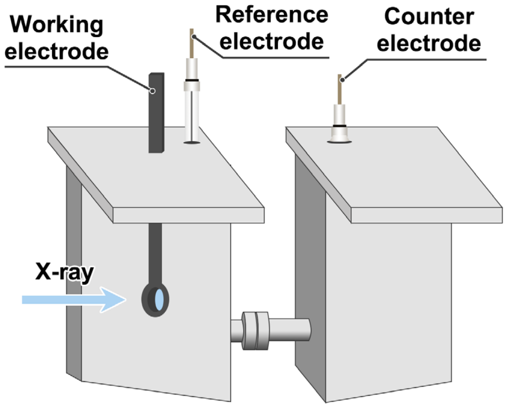

Supplementary Figure 16. Schematic illustration of the operando X-ray absorption spectroscopy (XAS) testing setup.

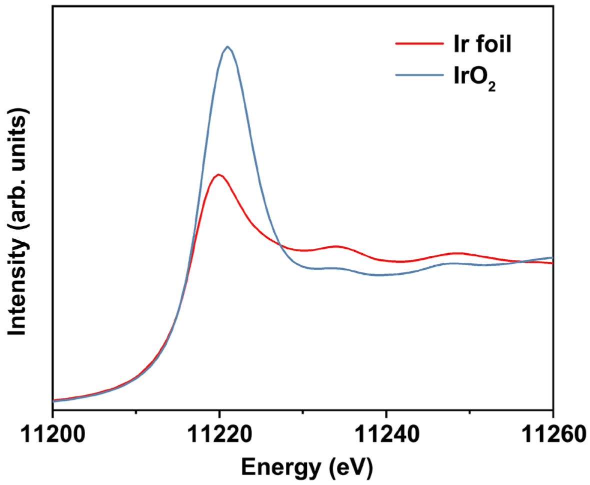

Supplementary Figure 17. Reference Ir L_3-edge XANES spectra of Ir foil and IrO_2.

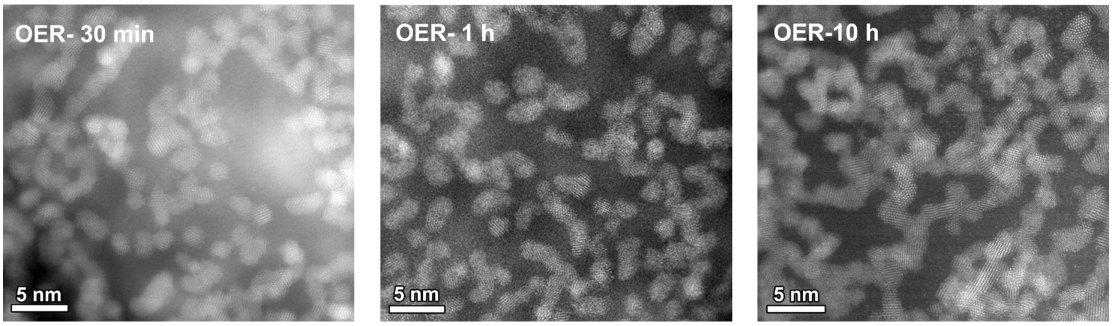

Supplementary Figure 18. The aberration-corrected HAADF-STEM images of OER-30 min, OER-1 h and OER-10 h.

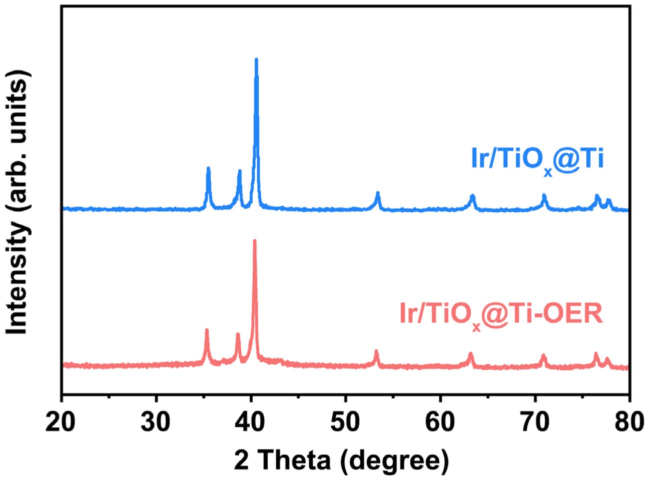

Supplementary Figure 19. XRD patterns of Ir/TiO$_x$@Ti and Ir/TiO$_x$@Ti after 10 hours of OER.

The XRD pattern after catalysis reveals no detectable alterations compared to the pristine sample, retaining only metallic Ti diffraction peaks while lacking IrO_2 signals (attributed to Ir's small-size effect). This demonstrates XRD's inherent limitation in resolving nanoscale structural evolution, thereby necessitating atomic-resolution characterization techniques like HAADF-STEM to explicitly reveal the metallic Ir-to-IrO_2 transformation pathway.

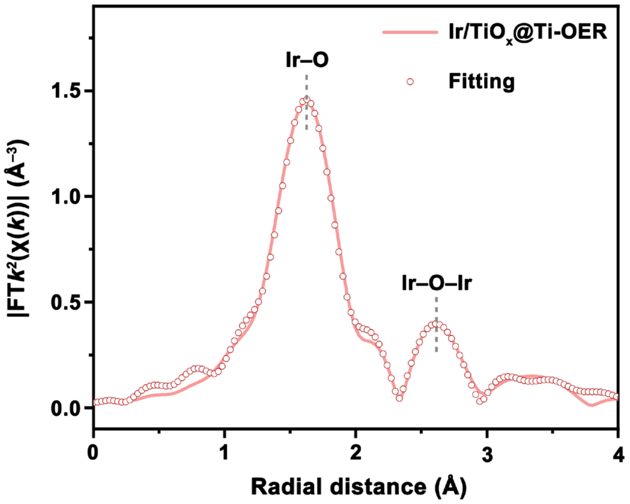

Supplementary Figure 20. Ir L_3-edge EXAFS spectra in R space of Ir/TiO$_x$@Ti after 10 hours of catalysis.

Our analysis confirms that Ir is fully converted to crystalline IrO_2 after 10 hours of catalytic operation. This complete oxidation is facilitated by the promoting effect of the TiO$_x$@Ti support, as discussed in relation to Fig. 4. Critically, the EXAFS R-space data for Ir/TiO$_x$@Ti after 10 hours of catalysis shows the complete absence of metallic Ir–Ir bonds (**Supplementary Figure 20** and **Supplementary Table 2**). This provides direct structural evidence for the full conversion of Ir to the oxide state (IrO_2).

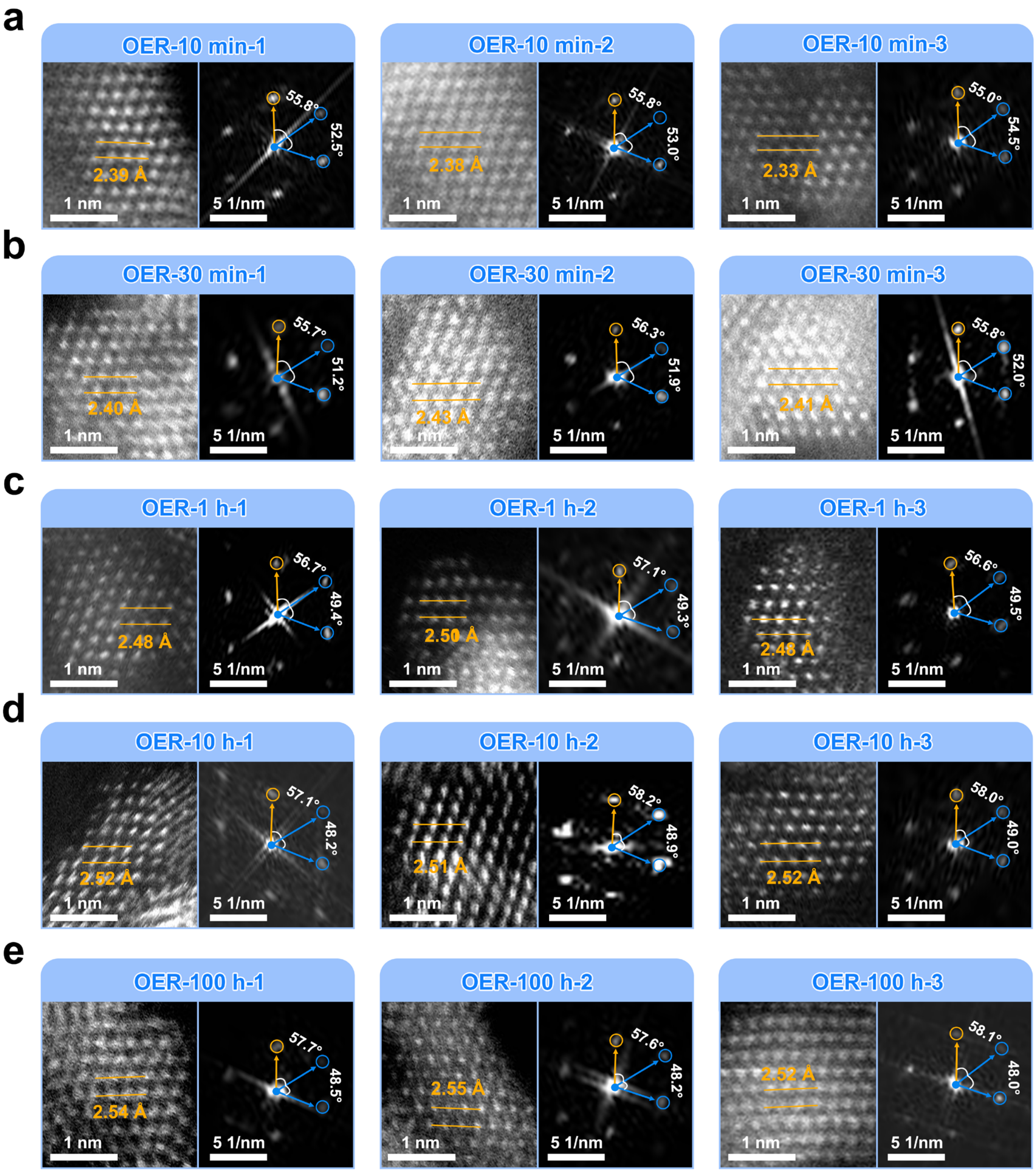

Supplementary Figure 21. Additional replicates of high-resolution HAADF-STEM and fast Fourier transform images for each post-catalysis sample, including: (a) OER-10 min, (b) OER-30 min, (c) OER-1 h, (d) OER-10 h, and (e) OER-100 h.

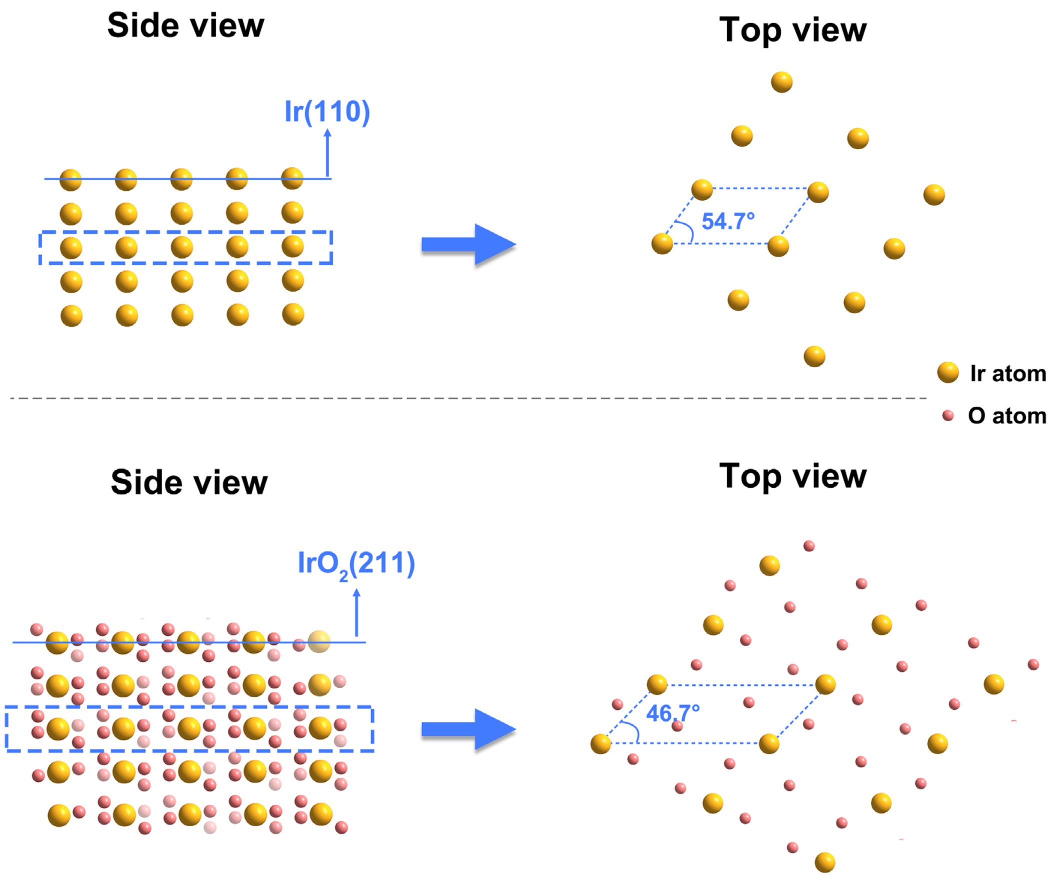

Supplementary Figure 22. Schematic diagram of structural similarity between Ir(110) and IrO_2(211).

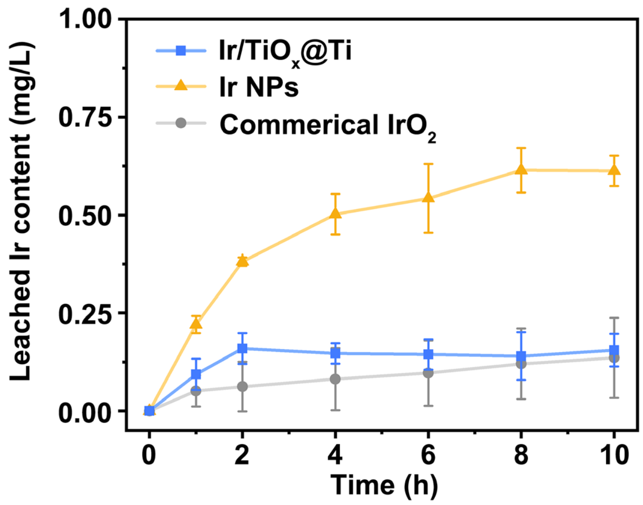

Supplementary Figure 23. The content of iridium leached into the electrolyte in the presence of Ir/TiO_x@Ti and Ir NPs and commercial IrO_2 as the electrocatalysts during the 10-hour OER catalysis. Error bars are drawn based on the standard deviations of three measurements.

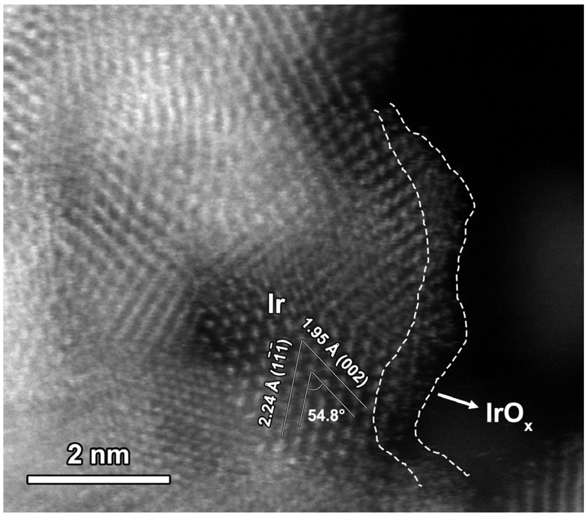

Supplementary Figure 24. HAADF-STEM image of Ir NPs after OER catalysis for 10 hours.

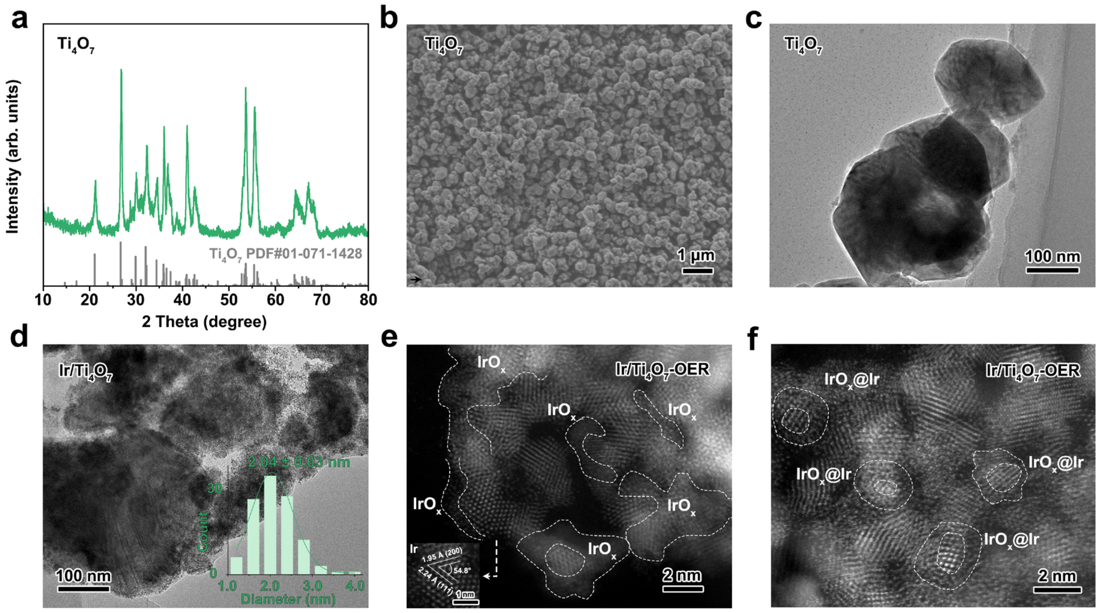

Supplementary Figure 25. (a) XRD pattern and (b) SEM image and (c) TEM image of Ti_4O_7. (d) TEM image of Ir/Ti_4O_7. The inset displays the size distribution histogram obtained from 110 particles (e, f) HAADF-STEM images of Ir/Ti_4O_7 after OER catalysis for 10 hours. (e) The inset shows the inside lattice fringes of a single iridium nanoparticle.

Ti_4O_7 nanoparticles are synthesized via an optimized solid-state reaction between Ti and TiO_2 (details in Methods), with their XRD pattern (**Supplementary Figure 25a**) confirming the formation of phase-pure Ti_4O_7. SEM and TEM images (**Supplementary Figures 25b** and **25c**) reveal Ti_4O_7 particles with an average size of ~200 nm. Subsequently, Ir/Ti_4O_7 was synthesized under identical conditions to those used for Ir/TiO_x@Ti. TEM imaging (**Supplementary Figure 25d**) demonstrates the successful deposition of ~2 nm Ir nanoparticles uniformly dispersed on the Ti_4O_7 surface. HAADF-STEM characterization of Ir/Ti_4O_7 after 10-hour OER catalysis (**Supplementary Figures 25e** and **25f)** further reveals significant surface amorphization of the Ir nanoparticles without crystalline IrO_2 transformation. This starkly contrasts with the bulk crystallization observed in Ir/TiO_x@Ti, unequivocally highlighting the unique role of the amorphous TiO_x@Ti support in driving the Ir-to-IrO_2 phase transition.

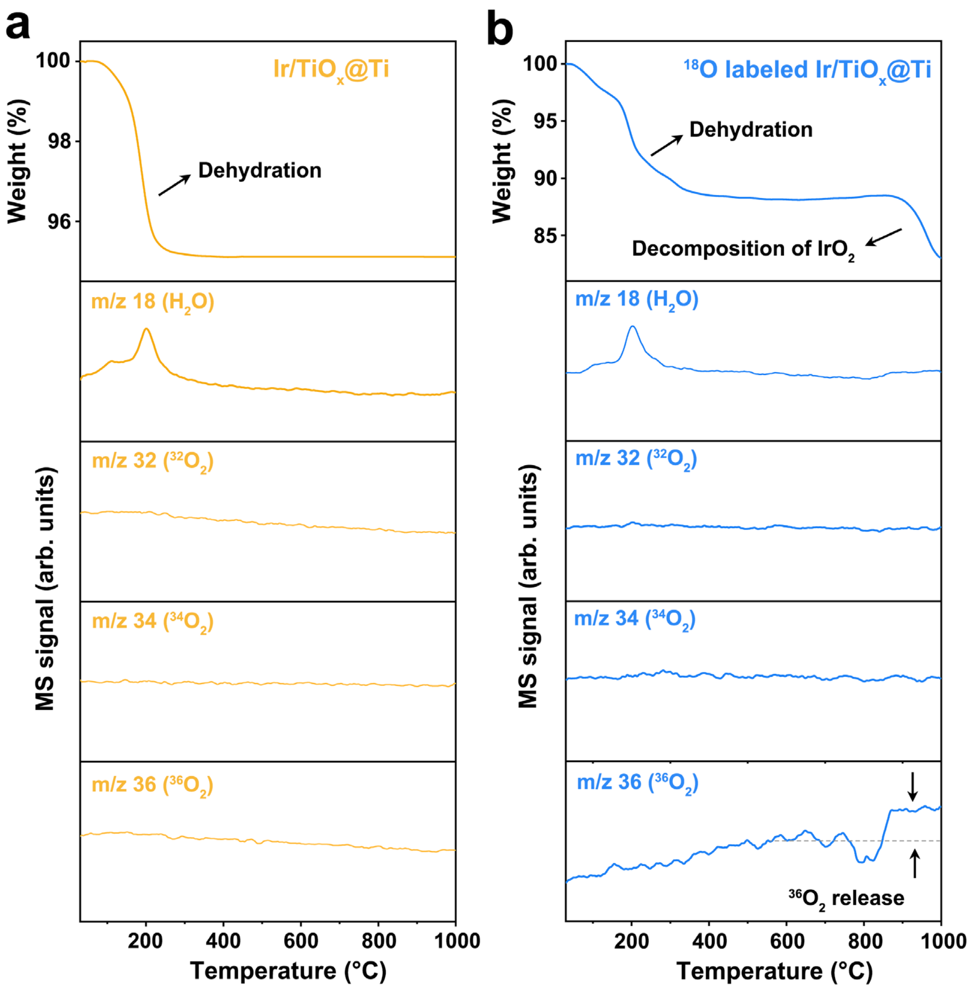

Supplementary Figure 26. TG-MS of (a) Ir/TiO_x@Ti and (b) ^{18}O-labeled Ir/TiO_x@Ti.

The pristine Ir/TiO_x@Ti exhibited a single weight loss at ~200°C, corresponding to dehydration, as evidenced by the m/z = 18 signal. No significant changes in $^{32}O_2$, $^{34}O_2$, or $^{36}O_2$ signals were observed (**Supplementary Figures 26a**). In contrast, the ^{18}O-labeled Ir/TiO_x@Ti displayed a second weight loss at ~850°C, attributed to IrO_2 decomposition. Mass spectrometry revealed no notable $^{32}O_2$ or $^{34}O_2$ signals, but a sharp increase in $^{36}O_2$ intensity coincided with IrO_2 decomposition (**Supplementary Figures 26b**). This unambiguously confirms that the oxygen incorporated into the Ir lattice originated exclusively from water, ruling out oxygen migration from the TiO_x@Ti support.

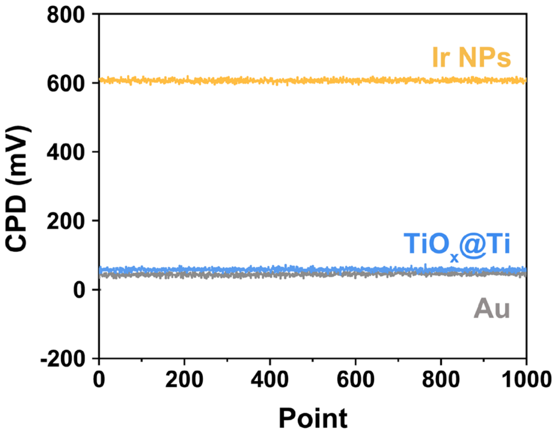

Supplementary Figure 27. Kelvin probe measurements reveal the contact potential difference (CPD) between samples (TiO_x@Ti and Ir NPs) and Au reference sample (φ_{Au} = 4.8 eV) in air. Thus, the work functions of TiO_x@Ti and Ir NPs are determined to be 4.8 eV and 5.4 eV, respectively.

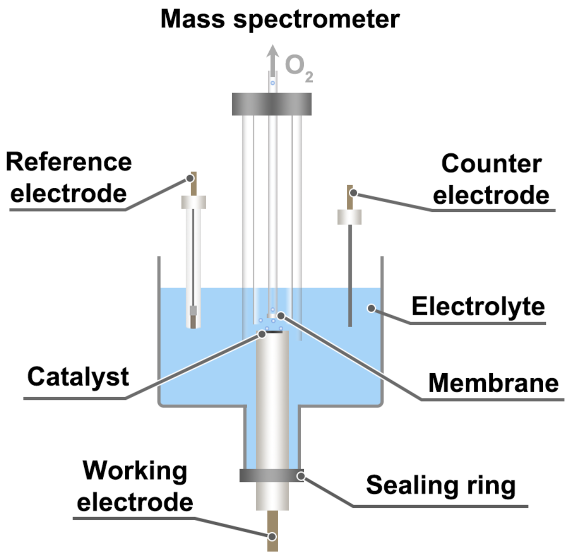

Supplementary Figure 28. Schematic illustration of the in situ electrochemical differential mass spectrometry (DEMS) testing setup.

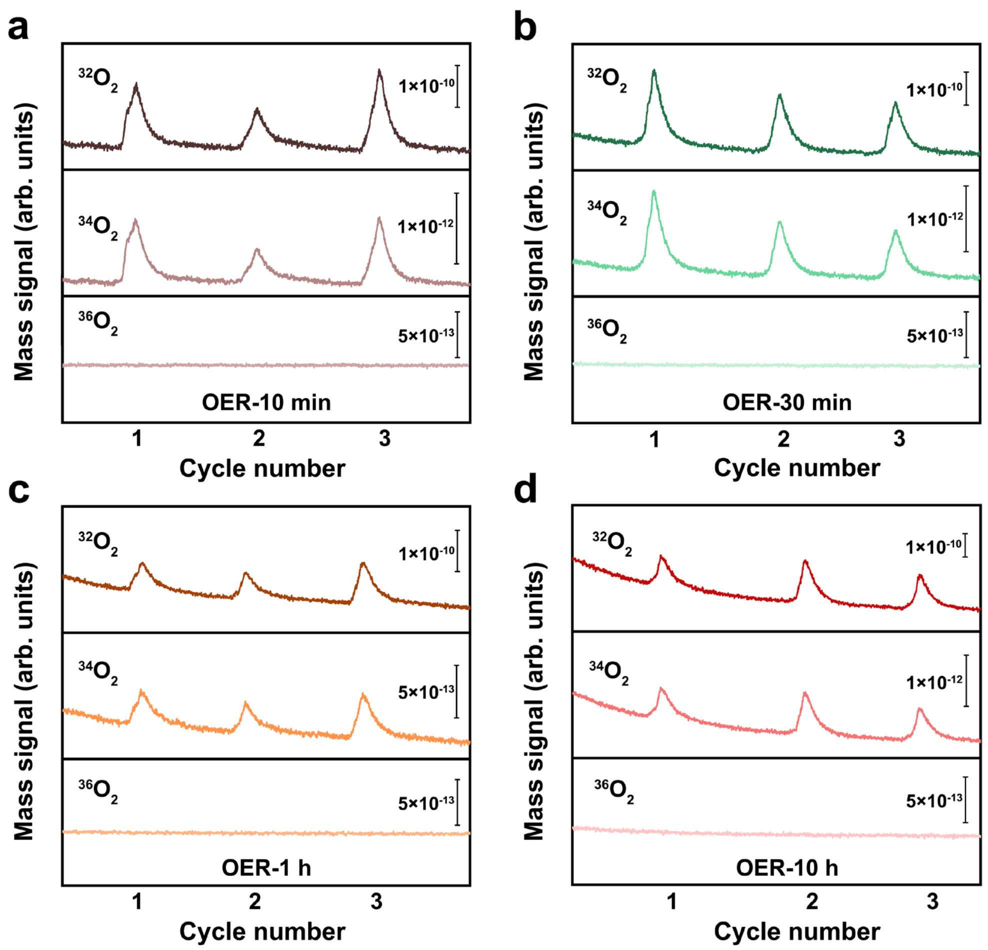

Supplementary Figure 29. Mass signals of $^{32}O_2$, $^{34}O_2$ and $^{36}O_2$ for ^{18}O-labeled Ir/TiO_x@Ti sample oxidized for (a) 10 minutes, (b) 30 minutes, (c) 1 hours and (d) 10 hours under OER conditions.

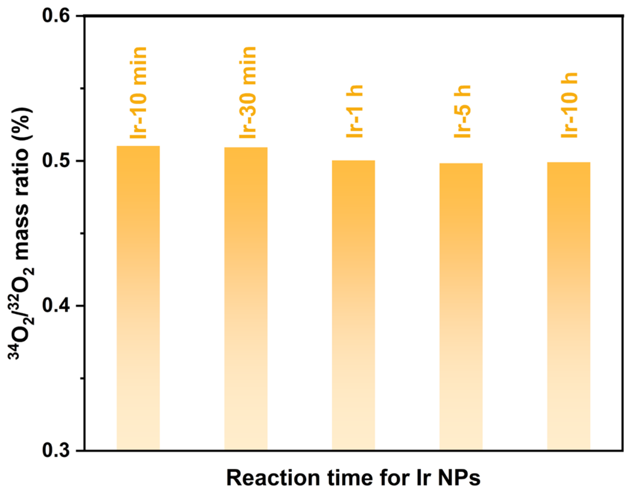

Supplementary Figure 30. Mass ratios of $^{34}O_2$ to $^{32}O_2$ for Ir NPs oxidized for different times under OER conditions.

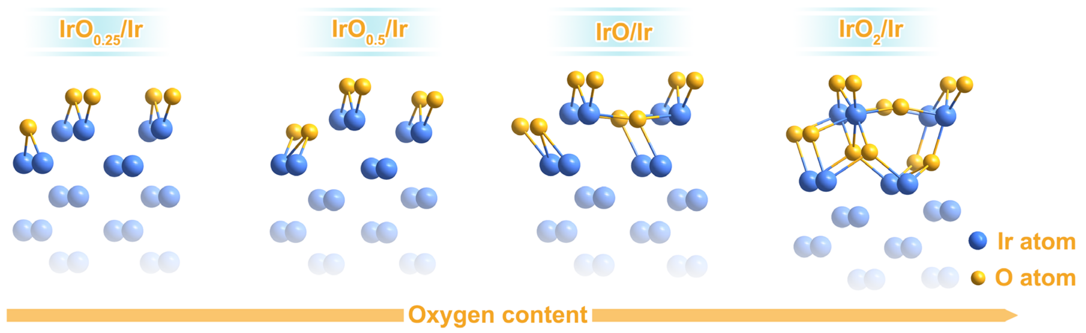

Supplementary Figure 31. The structural models of Ir with different oxygen contents used in theoretical calculations, including $IrO_{0.25}$/Ir, $IrO_{0.5}$/Ir, IrO/Ir and IrO_2/Ir.

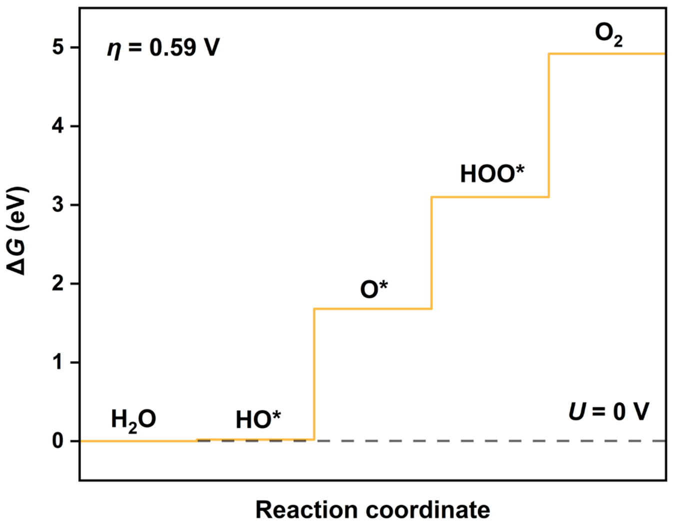

Supplementary Figure 32. Gibbs free energy diagram for OER of IrO_2(110) surface at external bias $U = 0$ V. The calculated overpotential η is 0.59 V at $U = 1.23$ V.

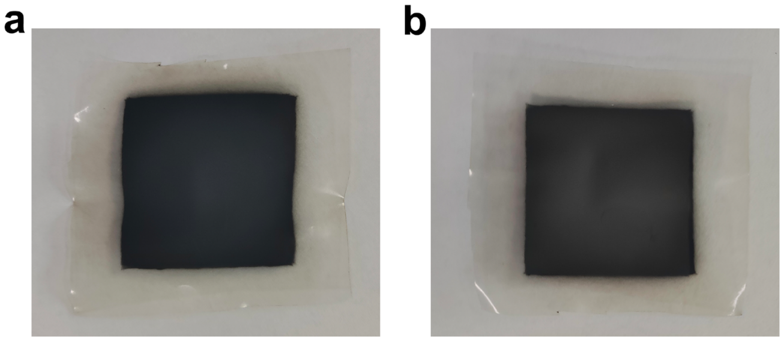

Supplementary Figure 33. Digital photographs of CCM (5 cm^2) for (a) Pt/C at cathode and (b) $Ir/TiO_x@Ti$ at anode.

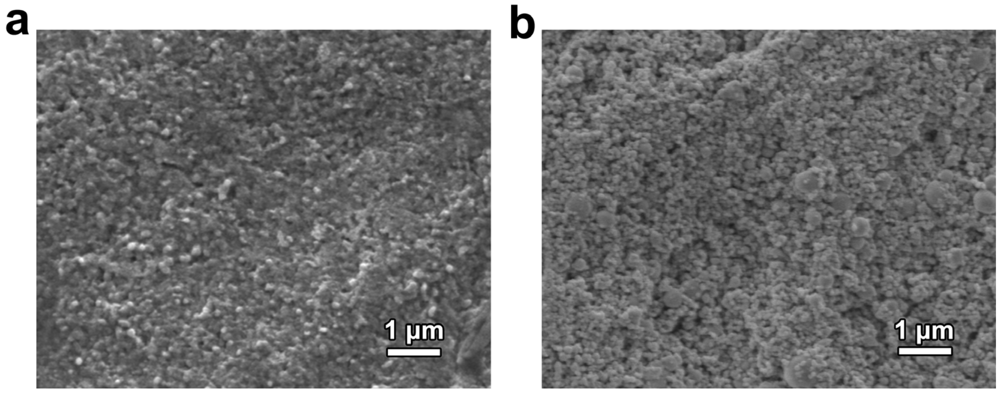

Supplementary Figure 34. SEM images of CCM for (a) Pt/C at cathode and (b) $Ir/TiO_x@Ti$ at anode.

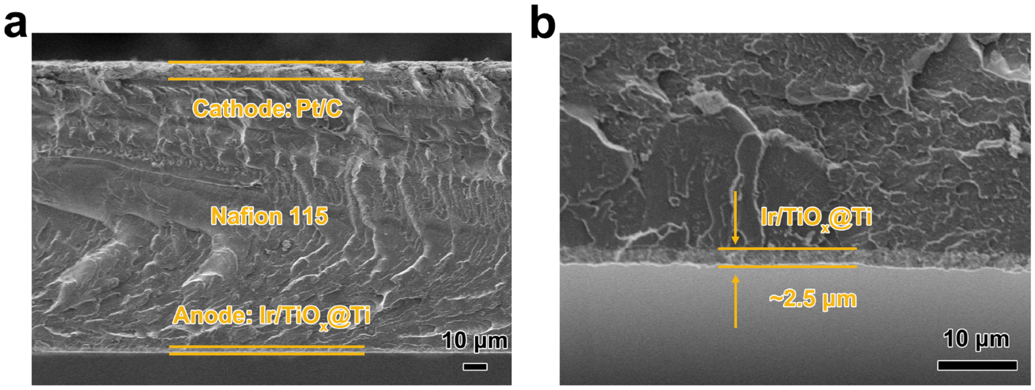

Supplementary Figure 35. (a) Cross-section SEM image of CCM employing Pt/C as cathodic catalyst layer and Ir/TiO_x@Ti as anodic catalyst layer. (b) Higher resolution cross-section SEM image focusing on the anodic Ir/TiO_x@Ti catalyst layer.

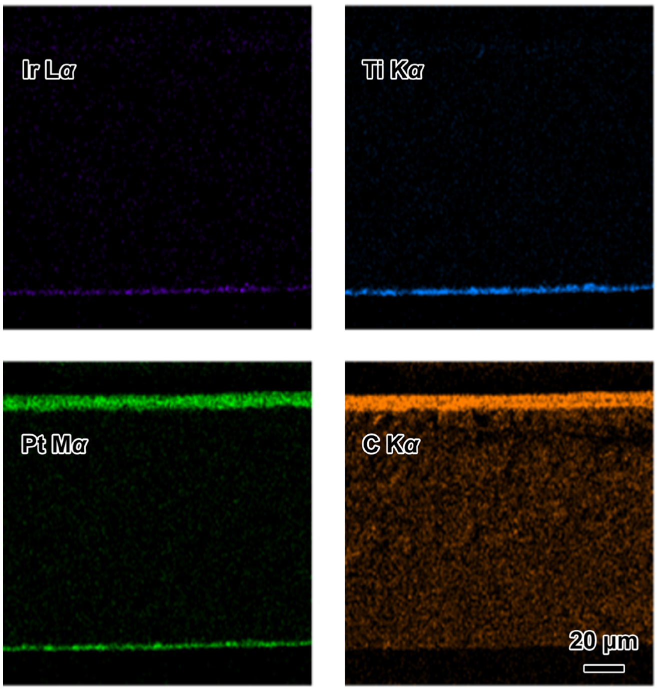

Supplementary Figure 36. Elemental mapping of CCM at the cross-section. Note: The energy position of the Ir M*α* peak is very similar to that of the Pt M*α* peak, which makes it difficult to distinguish the two elements in the elemental mapping shown at the bottom left.

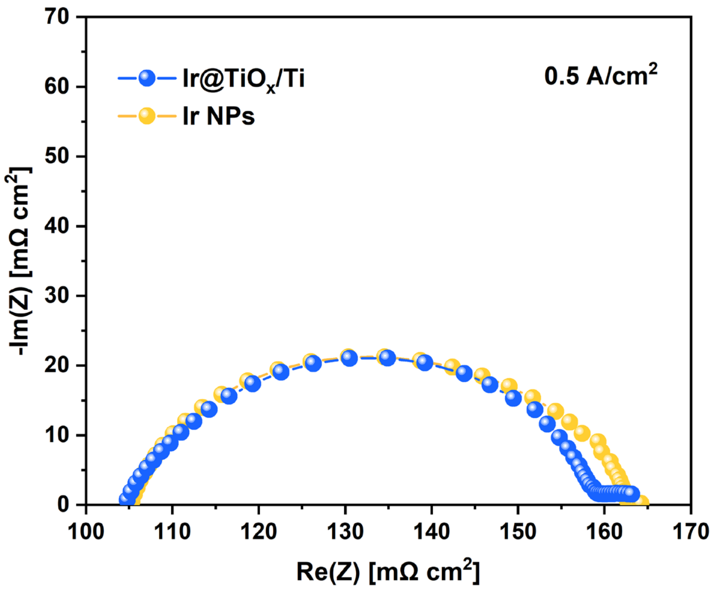

Supplementary Figure 37. The result of EIS to obtain high-frequency resistance (HFR).

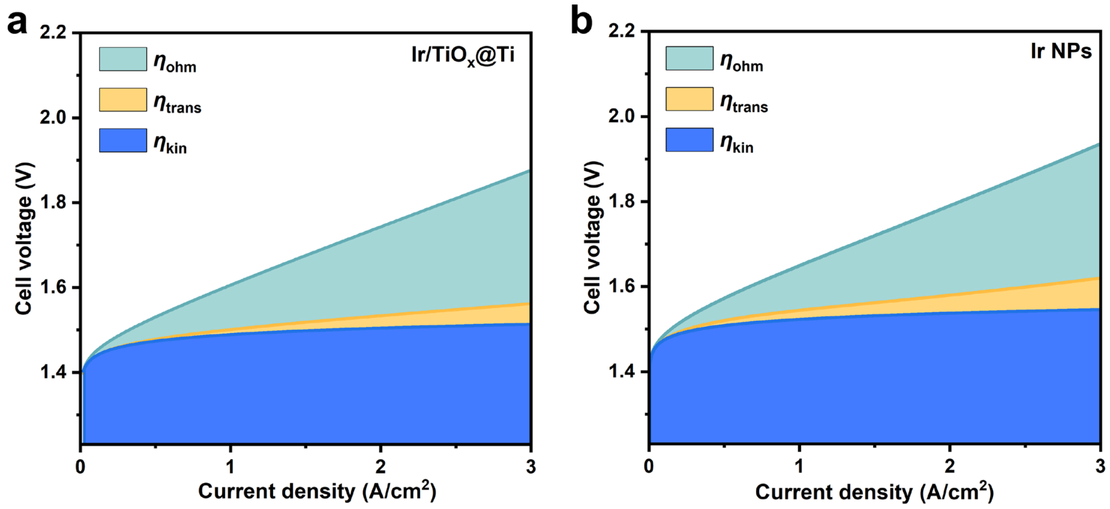

Supplementary Figure 38. The breakdowns of voltage losses for (a) Ir/TiO_x@Ti-based and (b) Ir NPs-based cells.

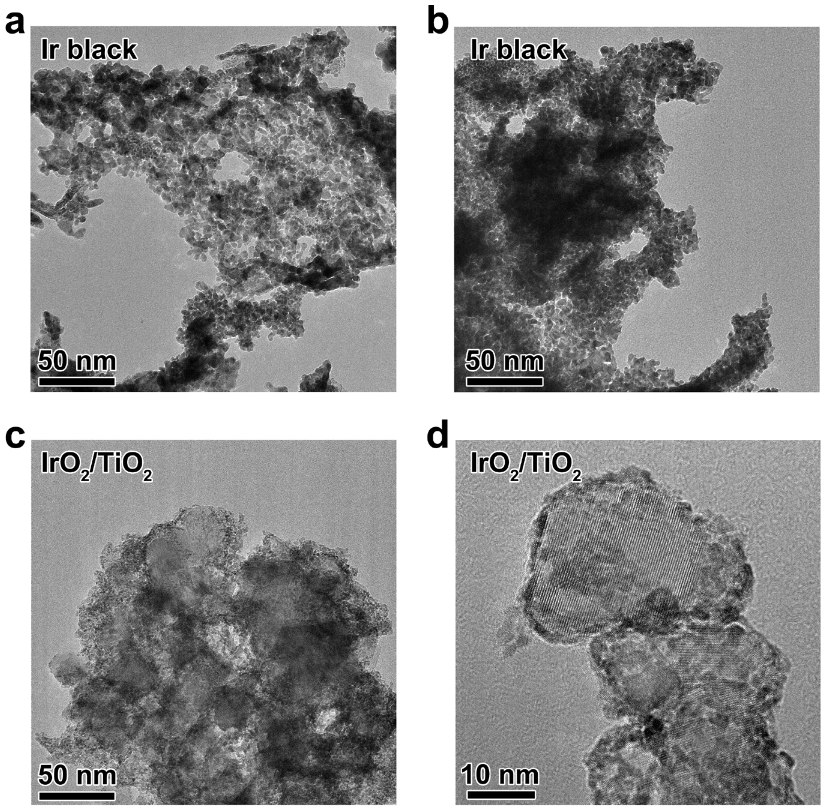

Supplementary Figure 39. TEM images for (a, b) commercial Ir black and (c, d) IrO_2/TiO_2 (Heraeus S60).

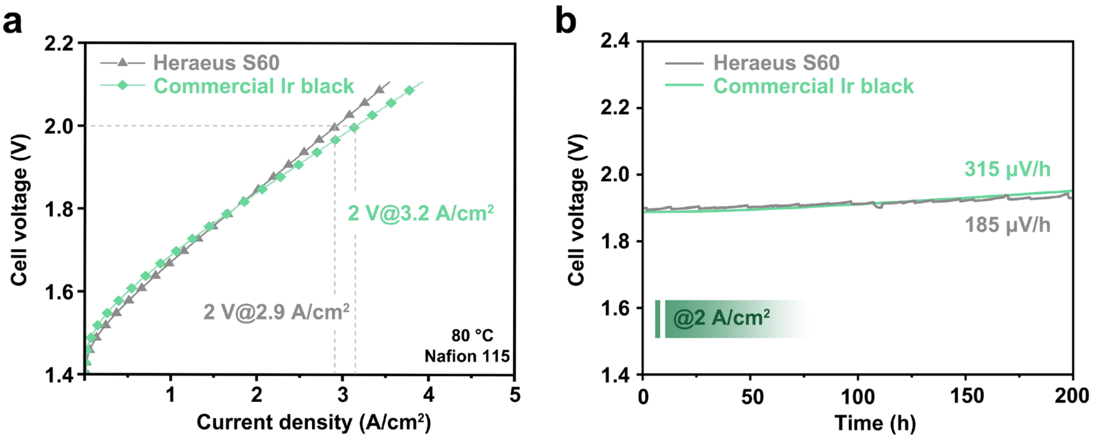

Supplementary Figure 40. (a) Steady-state polarization curves of PEMWEs using commercial Ir black and Heraeus S60 (IrO_2/TiO_2) as anode catalysts with Ir loading of 0.3 mg/cm^2 (b) Chronopotentiometry curves of PEMWEs using commercial Ir black and Heraeus S60 anode operated at 2.0 A/cm^2 current density with Ir loading of 0.3 mg/cm^2.

Supplementary Table 1. Comparison of OER stability of Ir/TiO_x@Ti with representative supported Ir electrocatalysts.

Catalyst	Electrolyte	Stability	Reference
Ir/TiO_x@Ti	0.1 M $HClO_4$	1.52 V@1744 h	This work
Ir NPs	0.1 M $HClO_4$	1.75 V @50 h	This work
Ir/WO_{3-x}	0.5 M H_2SO_4	1.62 V@105 h	Ref. 3
Ir/TiO_2	0.5 M H_2SO_4	1.47 V@100 h	Ref. 4
Ir/ATO	0.05 M H_2SO_4	1.52 V@15 h	Ref. 5
Ir/$SrIrO_3$	0.5 M H_2SO_4	1.50 V@25 h	Ref. 6
Ir/Nb_2O_{5-x}	0.5 M H_2SO_4	1.53 V@100 h	Ref. 7
Ir/Ta_2O_5	0.1 M $HClO_4$	1.58 V@3 h	Ref. 8

Supplementary Table 2. EXAFS fitting parameters at the Ir L_3-edge for Ir/TiO_x@Ti and Ir NPs.

Sample	Shell	CN^a	R(Å)^b	σ^2 (Å^2·10^{-3})^c	ΔE_0(eV)^d	R factor
Ir/TiO_x@Ti at OCP	Ir–O	3.75±0.65	2.13±0.04	0.008±0.007	8.92±2.14	0.0095
	Ir–Ir	6.38±0.23	2.68±0.03	0.016±0.009		
Ir/TiO_x@Ti at 1.25 V	Ir–O	4.32±0.78	1.99±0.02	0.030±0.018	9.51±1.79	0.0021
	Ir–Ir	5.01±0.75	2.58±0.04	0.021±0.017		
Ir/TiO_x@Ti at 1.35 V	Ir–O	5.37±1.12	1.93±0.03	0.021±0.008	8.14±1.84	0.0071
	Ir–Ir	4.78±0.85	2.48±0.03	0.016±0.005		
Ir/TiO_x@Ti at 1.45 V	Ir–O	5.57±0.32	1.91±0.07	0.004±0.004	8.06±2.62	0.0033
	Ir–Ir	4.47±0.85	2.47±0.04	0.008±0.017		
Ir NPs at OCP	Ir–O	2.84±0.48	2.05±0.02	0.019±0.009	7.85±2.51	0.0023
	Ir–Ir	5.91±0.34	2.73±0.05	0.011±0.010		
Ir NPs at 1.25 V	Ir–O	3.06±0.35	1.95±0.08	0.024±0.008	7.15±3.11	0.0099
	Ir–Ir	4.96±0.95	2.69±0.05	0.008±0.007		
Ir NPs at 1.35 V	Ir–O	3.72±0.75	1.89±0.06	0.006±0.002	7.71±2.58	0.0027
	Ir–Ir	4.56±0.52	2.67±0.02	0.011±0.010		
Ir NPs at 1.45 V	Ir–O	4.13±0.78	1.90±0.08	0.028±0.008	7.35±1.87	0.0038
	Ir–Ir	4.55±0.10	2.67±0.04	0.017±0.010		
Ir/TiO_x@Ti -OER	Ir–O	6.13±0.38	2.12±0.05	0.012±0.008	8.27±1.38	0.0038
	Ir–O–Ir	1.12±1.11	3.07±0.10	0.025±0.004		

Note: ^{a}CN, coordination number; ^{b}R, the distance between absorber and backscatter atoms; $^c\sigma^2$, Debye-Waller factor to account for both thermal and structural disorders; $^d\Delta E^0$, inner potential correction; R factor indicates the goodness of the fit. S_0^2 is fixed to 0.815, according to the experimental EXAFS fit of Ir foil by fixing CN as the known crystallographic value. Fitting range: k(Å^{-1}): 2.0–12.0; R(Å): 1.0–3.5; fitting space: R space. A reasonable range of EXAFS fitting parameters: $0.800 < S_0^2 < 1.000$; CN > 0; $\sigma^2 > 0$ Å^2; $|\Delta E_0| < 10$ eV; R factor < 0.02.

Supplementary Table 3. Δ*G* of AEM and LOM pathways for $IrO_{0.25}$/Ir, $IrO_{0.5}$/Ir, IrO/Ir, and IrO_2/Ir at U = 1.23 V.

Sample	pathway	ΔG_1 (eV)	ΔG_2 (eV)	ΔG_3 (eV)	ΔG_4 (eV)	ΔG_5 (eV)	ΔG_6 (eV)	ΔG_{max} (eV)
$IrO_{0.25}$/Ir	AEM	–0.59	–0.58	1.21	–0.05	–	–	1.21
	LOM	–0.59	–0.58	0.43	0.05	0.57	0.12	0.57
$IrO_{0.5}$/Ir	AEM	–0.79	–0.16	0.85	0.09	–	–	0.85
	LOM	–0.79	–0.16	1.22	–0.39	0.53	–0.42	1.22
IrO/Ir	AEM	–0.65	–0.17	0.81	0.01	–	–	0.81
	LOM	–0.65	–0.17	1.81	–0.67	–0.53	0.21	1.81
IrO_2/Ir	AEM	–1.25	0.32	0.41	0.53	–	–	0.53
	LOM	–1.25	0.32	2.46	–0.02	–1.29	–0.21	2.46

Supplementary Table 4. Reported performances of PEMWE anode catalysts and corresponding parameters.

Catalysis	Membrane	Activity (A/cm^2@ 1.79 V)	Ir loading (mg/cm^2)	Ir-specific power (kW/g_{Ir})	Reference
Ir/TiO_x@Ti	N115	2.35	0.3	14.2	This work
Ir/TiO_2-MoO_x	N115	1.44	0.5	5.2	Ref. 9
Ir/Ta_2O_5	N115	1.52	0.3	9.1	Ref. 8
Ir/Nb_2O_{5-x}	N115	2.45	1.8	2.4	Ref. 7
Ir/$W_xTi_{1-x}O_2$	N115	2.2	0.4	9.8	Ref. 10
Ir-ND/ATO	NR212	1.48	1	2.7	Ref. 5
Ir/B_4C	N115	1.92	0.5	6.9	Ref. 11
IrO_2@TaO_x@TaB	N115	2.2	0.26	15.2	Ref. 12
IrO_2@TiN_{1+x}	N117	1.47	1.2	2.2	Ref. 13
IrO_2/TiO_2	N115	2	0.3	11.9	Ref. 14
IrO_2/Ti_4O_7	N115	1.27	0.24	9.4	Ref. 15
IrO_x/Zr_2ON_2	N115	1.36	0.4	6.1	Ref. 16

References for SI

1. Yang, B. *et al.* Flatband λ-Ti_3O_5 towards extraordinary solar steam generation. *Nature* **622**, 499-506 (2023).
2. Li, X. *et al.* Magneli phase Ti_4O_7 electrode for oxygen reduction reaction and its implication for zinc-air rechargeable batteries. *Electrochim. Acta* **55**, 5891-5898 (2010).
3. Ma, X. *et al.* Oxygen-vacancy-rich tungsten oxide boosted ultrasmall iridium nanoparticles for acidic oxygen evolution. *Int. J. Hydrogen Energy* **48**, 36776-36783 (2023).
4. Chen, J., Jayabal, S., Geng, D. & Hu, X. Monolayer Iridium Nanoparticles Coated TiO_2 Core-Shell Architecture as Efficient Oxygen Evolution Reaction Electrocatalyst. *ChemistrySelect* **6**, 9134-9138 (2021).
5. Oh, H.-S., Nong, H. N., Reier, T., Gliech, M. & Strasser, P. Oxide-supported Ir nanodendrites with high activity and durability for the oxygen evolution reaction in acid PEM water electrolyzers. *Chem. Sci.* **6**, 3321-3328 (2015).
6. Zhao, L. *et al.* Partial Exsolution Enables Superior Bifunctionality of Ir@$SrIrO_3$ for Acidic Overall Water Splitting. *Adv. Sci.* **11**, 2309750 (2024).
7. Shi, Z. *et al.* Enhanced Acidic Water Oxidation by Dynamic Migration of Oxygen Species at the Ir/Nb_2O_5−Catalyst/Support Interfaces. *Angew. Chem. Int. Ed.* **61**, e202212341 (2022).
8. Baik, C. *et al.* Electron-rich Ir nanostructure supported on mesoporous Ta_2O_5 for enhanced activity and stability of oxygen evolution reaction. *J. Power Sources* **575**, 233174 (2023).
9. Kim, E.-J. *et al.* Stabilizing role of Mo in TiO_2-MoO_x supported Ir catalyst toward oxygen evolution reaction. *Appl. Catal., B* **280**, 119433 (2021).
10. Zhao, S., Stocks, A., Rasimick, B., More, K. & Xu, H. Highly Active, Durable Dispersed Iridium Nanocatalysts for PEM Water Electrolyzers. *J. Electrochem. Soc.* **165**, F82 (2018).
11. Islam, J. *et al.* Enhancing the activity and durability of iridium electrocatalyst supported on boron carbide by tuning the chemical state of iridium for oxygen evolution reaction. *J. Power Sources* **512**, 230506 (2021).
12. Wang, Y. *et al.* Supported IrO_2 Nanocatalyst with Multilayered Structure for Proton Exchange Membrane Water Electrolysis. *Adv. Mater.* **36**, 2407717 (2024).
13. Wang, S. *et al.* Defects tailoring IrO_2@TiN_{1+x} nano-heterojunctions for superior water oxidation activity and stability. *Mater. Chem. Front.* **5**, 8047-8055 (2021).
14. Yang, C. *et al.* Surface hydroxylation engineering to boost oxygen evolution reaction on IrO_2/TiO_2 for PEM water electrolyzer. *Appl. Catal. B: Environ.* **358**, 124462 (2024).
15. Qin, Y. *et al.* Interface-Engineering Strategy for Boosting Low-Ir Catalytic Water Oxidation Using a Conductive Ti_4O_7 Support. *ACS Catal.* **14**, 13915-13926 (2024).
16. Lee, C. *et al.* Catalyst-Support Interactions in Zr_2ON_2-Supported IrO_x Electrocatalysts to Break the Trade-Off Relationship Between the Activity and Stability in the Acidic Oxygen Evolution Reaction. *Adv. Funct. Mater.* **33**, 2301557 (2023).
