# Supplementary material for: Support-tuned iridium reconstruction with crystalline phase dominating acidic oxygen evolution
Source: Nat Commun. 2025 Sep 1;16:8164. doi: 10.1038/s41467-025-63541-9 (PMC12402471; doi:10.1038/s41467-025-63541-9)
Supplement: Supplementary file 2 — Description of Additional Supplementary Files [file 41467_2025_63541_MOESM2_ESM.pdf]

**Description of Additional Supplementary Files**

File name: Supplementary Data 1

Description:

(1) The Figure 4a folder contains optimized structure files for various surface coverages (1 ML H, 1/2 ML OH + 1/2 ML $H_2O$, 1 ML OH, 1 ML O) and the bare Ir surface, used to compute the Pourbaix diagram for Ir surfaces in Figure 4a.

(2) The Figure 6e folder contains optimized structure files for all intermediates involved in both the AEM and LOM mechanisms on Ir surface models with different subsurface oxygen contents, as presented in Figure 6e.

(3) The Figure 6f folder contains optimized structure files for all intermediates involved in the AEM mechanism at both Ir1 and Ir2 sites on the $IrO_2$(211) surface, used for calculations in Figure 6f.

(4) The Figure 6f folder contains optimized structure files for all intermediates involved in the AEM mechanism at the Ir site on the rutile $IrO_2$(110) surface, used for calculations in Figure 6f.
